# The potential role of long noncoding RNAs in primary open-angle glaucoma

**DOI:** 10.1007/s00417-021-05279-w

**Published:** 2021-07-10

**Authors:** Feng Zhang, Yang Zhao, Mengdan Cao, Xu Jia, Zheng Pan, Dengming Zhou, Ke Liu, Xuanchu Duan

**Affiliations:** 1 Department of Ophthalmology, The Third Xiangya Hospital, Central South University, Changsha, Hunan Province China; 2 Aier School of Ophthalmology, Central South University, Changsha, Hunan Province China; 3 Changsha Aier Eye Hospital, Changsha, Hunan Province China; 4 Department of Ophthalmology, The Second Xiangya Hospital, Central South University, Changsha, Hunan Province China; 5 Affiliated Hospital of Guizhou Medical University, Guiyang, Guizhou Province China

**Keywords:** Long noncoding RNA, Primary open-angle glaucoma, Trabecular meshwork

## Abstract

**Purpose:**

To identify the potential genes in human trabecular meshwork (TM) related to primary open-angle glaucoma (POAG).

**Methods:**

First, long noncoding RNA (LncRNA) and mRNA expression profiles in TM samples from 4 control subjects and 4 POAG patients were accessed by microarray analyses. Then, twenty lncRNAs were validated by real-time quantitative PCR in the same samples from microarray analyses. Finally, eight highly expressed lncRNAs were further tested by real-time quantitative PCR in TM from 8 normal controls and 19 POAG patients. Expression data were normalized and analyzed using the R software. Pathway analyses were performed by Gene Ontology (GO) and Kyoto Encyclopedia of Genes and Genomes (KEGG) analysis.

**Results:**

A total of 2179 lncRNAs and 923 mRNAs in the TM of POAG patients were significantly upregulated, and 3111 lncRNAs and 887 mRNAs were significantly downregulated. ENST00000552367, ENST00000582505, ENST00000609130, NR_029395, NR_038379, and ENST00000586949 expression levels were significantly higher in the TM from a different cohort of POAG patient than normal controls.

**Conclusion:**

ENST00000552367, ENST00000582505, ENST000006091- 30, NR_029395, NR_038379, and ENST00000586949 may play essential roles in the development of POAG. 
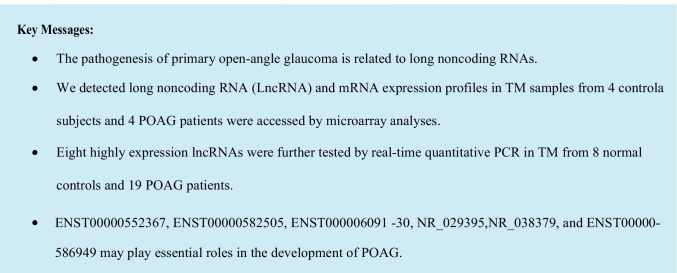

## Introduction

Glaucoma, after cataracts, is the most frequent cause of blindness worldwide [1], affecting more than 60 million people. Also, the number of primary open-angle glaucoma (POAG) patients is estimated to be 45 million around the world [2]. Recognized risk factors for POAG include elevated intraocular pressure (IOP) [3], genetic factors [4], environmental circumstances [5, 6], refractive error [7], and systemic diseases [8, 9].

With the discovery and study of noncoding RNAs which contain miRNAs, circular RNAs, and long noncoding RNAs (lncRNAs), the relationship between ncRNAs and diseases has raised concern recently [10]. LncRNAs (ncRNAs > 200 nucleotides in length) have long been regarded as junk RNAs. Recently, however, lncRNAs have been shown to play key roles in a variety of cellular processes through interaction with the main component proteins in gene regulatory systems [11]. Currently, lncRNAs were shown to take part in the biomarker [12], development, and progression of glaucoma [13].

In our study, we performed microarray assays to obtain an overview of the expression profiles of various lncRNAs and mRNAs in the trabecular meshwork of POAG patients and normal subjects. Disease-related lncRNA profiles in the trabecular meshwork of POAG patients have been discovered. We found that ENST00000552367, ENST00000582505, ENST000006091- 30, NR_029395, NR_038379, and ENST00000586949 may play an essential role in the development of POAG.

## Materials and methods

### Procurement of trabecular meshwork

The study conforms to all tenets of the Declaration of Helsinki, and written informed consent was obtained from all subjects. This research was approved by the Ethics Committee of The Second Xiangya Hospital of Central South University (Changsha, China). All donated samples were obtained from The Second Xiangya Hospital.

Trabecular meshwork for test group was obtained from POAG patients who had uncontrolled IOP and accepted trabeculectomy surgery performed by one surgeon (XC. D). The inclusion criteria of POAG were the following: (1) age at POAG diagnosis older than 30 years, (2) glaucomatous optic nerve damage with associated visual field damage, and (3) exclude secondary glaucoma. All control TM tissue was obtained from donor eyes without glaucoma or glaucoma-associated condition.

### RNA isolation and qPCR

TRIzol Reagent (Invitrogen Life Technologies, Carlsbad, CA) was used to extract total RNA from the TM samples. The total RNA quantity and quality were measured by NanoDrop ND-1000. RNA integrity was assessed by standard denaturing agarose gel electrophoresis. Total RNA was also purified with RNeasy MinElute Cleanup Kit (Qiagen, Hilden, Germany) according to the manufacturer’s protocol. RNA was reverse transcribed into cDNA with the SuperScript™ III Reverse Transcriptase (Invitrogen, CA). Then, the cDNA was used for carrying out quantitative RT-PCR which was conducted by SYBR green expression master mix (Applied Biosystems, Inc., Foster City, CA, USA). The forward and reverse primer sequences are listed in Table 2. The △△CT method (2^−△△Ct^) was applied to calculate the relative differences between the control and POAG groups.

### Microarray analysis

TM RNA samples from 4 control subjects and 4 POAG patients for microarray analyses were extracted and the RNA integrity was tested by standard denaturing agarose gel electrophoresis, as described above. RNA sample labeling and array hybridization were performed according to the Agilent One-Color Microarray-Based Gene Expression Analysis Protocol (Agilent, Santa Clara, CA). Briefly, mRNA was purified from total RNA after removal of rRNA (mRNA-ONLY™ Eukaryotic mRNA Isolation Kit, Epicentre). Then, each sample was amplified and transcribed into fluorescent cRNA along the entire length of the transcripts without 3′ bias utilizing a random priming method (Arraystar Flash RNA Labeling Kit, Arraystar). The labeled cRNAs were purified by RNeasy Mini Kit (Qiagen). The concentration and specific activity of the labeled cRNAs (pmol Cy3/μg cRNA) were measured by NanoDrop ND-1000. One microgram of each labeled cRNA was fragmented by adding 5 μl 10 × blocking agent and 1 μl of 25 × fragmentation buffer, then the mixture was heated at 60 °C for 30 min; finally, 25 μl 2 × GE Hybridization buffer was added to dilute the labeled cRNA. Fifty microliters of hybridization solution was dispensed into the gasket slide and assembled to the lncRNA expression microarray slide. The slides were incubated for 17 h at 65 °C in an Agilent Hybridization Oven. The hybridized arrays were washed, fixed, and scanned using the Agilent DNA Microarray Scanner (part number G2505C).

Data were extracted through the Agilent Feature Extraction software (Agilent, Santa Clara, CA). All original data have been uploaded to Gene Expression Omnibus public database (https://www.ncbi.nlm.nih.gov/geo; GSE138125).

### GO and KEGG enrichment analysis

The Gene Ontology (GO) (http://www.geneontology.org) is a major bioinformatic tool to annotate genes and analyze biological process of these genes [14]. Kyoto Encyclopedia of Genes and Genomes (KEGG) (http://www.genome.jp/kegg) is a database resource for understanding high-level functions and biological systems from large-scale molecular datasets [15]. P < 0.05 was considered statistically significant.

### Statistical analysis

A train of data processing was performed through the R software package version 3.6.0 [16]. Numeric variables were compared using t-test. Results were expressed as means ± standard deviation. All statistical analyses were performed with GraphPad Prism 7 (GraphPad Software, USA). The results were considered significant if P < 0.05.

## Results

### Demographics and characteristics of POAG cases and controls

Our study included 23 human TMs from POAG patients and 12 healthy controls who donated their eyes after death from the Second Xiangya hospital, Central South University. The baseline characteristics of the subjects are summarized in Table 1. Subjects in the control and POAG groups were aged 49.83 ± 10.16 (mean ± SD) and 50.43 ± 9.72 years, respectively. The male percentage of the control and POAG groups was 58.33% and 43.48%, respectively.

**Table 1:** Clinical characteristics of subjects which trabecular meshwork was used in the study

Characteristics	Control (n = 12)	POAG (n = 23)
Age, mean ± SD, years	49.83 ± 10.16	50.43 ± 9.72
Sex, %		
Male Female	58.33 41.67	43.48 56.52

### Microarray expression profiling of lncRNAs and mRNAs in trabecular mesh from individual subjects

**Fig. 1:**
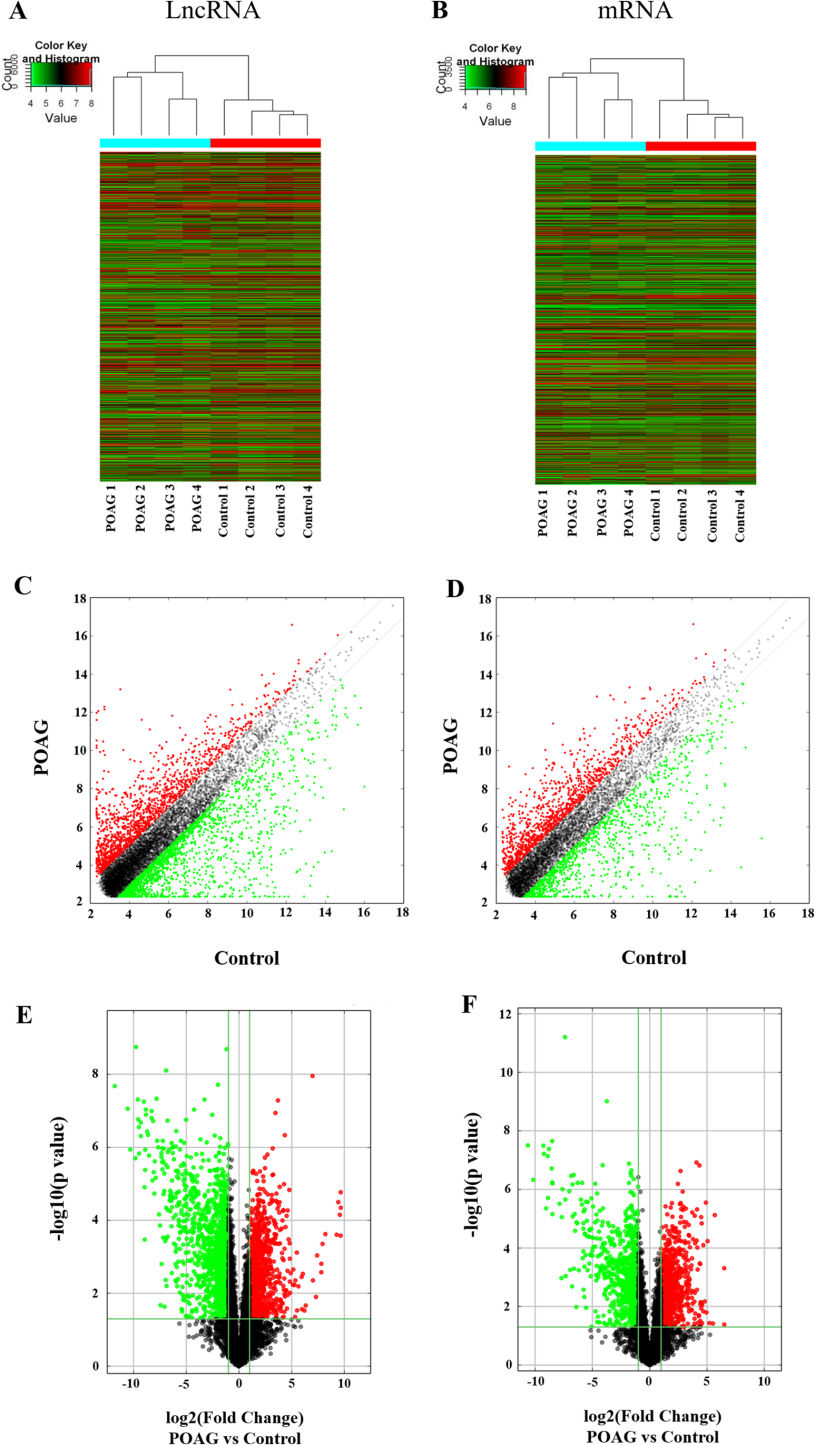
Microarray expression profiling of long noncoding RNAs (lncRNAs) and mRNAs in the trabecular meshwork (TM). (A) (B) Heat maps of lncRNA (A) and mRNA (B) microarray expression profiling in TM of normal controls and primary open-angle glaucoma (POAG) patients. (C) (D) Scatter plots of lncRNAs (C) and mRNAs (D) expression profile. (E) (F) Volcano plots of differentially expressed lncRNAs (E) and mRNAs (F) in TM between control group and POAG group. The vertical green lines and horizontal green line indicate cutoff lines for fold change and P values, respectively (fold change ≤ 0.5 or ≥ 2, and P ≤ 0.05)

To detect and identify differentially expressed lncRNAs and mRNAs in the trabecular meshwork of POAG and normal patients, tissue samples were collected and quantified by microarray assays (Fig. 1). A total of 2179 lncRNAs and 923 mRNAs were significantly upregulated (fold change ≥ 2, false discovery rate ≤ 0.05, P ≤ 0.05), and 3111 lncRNAs and 887 mRNAs were significantly downregulated (fold change ≥ 2, false discovery rate ≤ 0.05, P ≤ 0.05), in POAG patients compared with control subjects.

### GO and KEGG pathway enrichment analysis

**Fig. 2:**
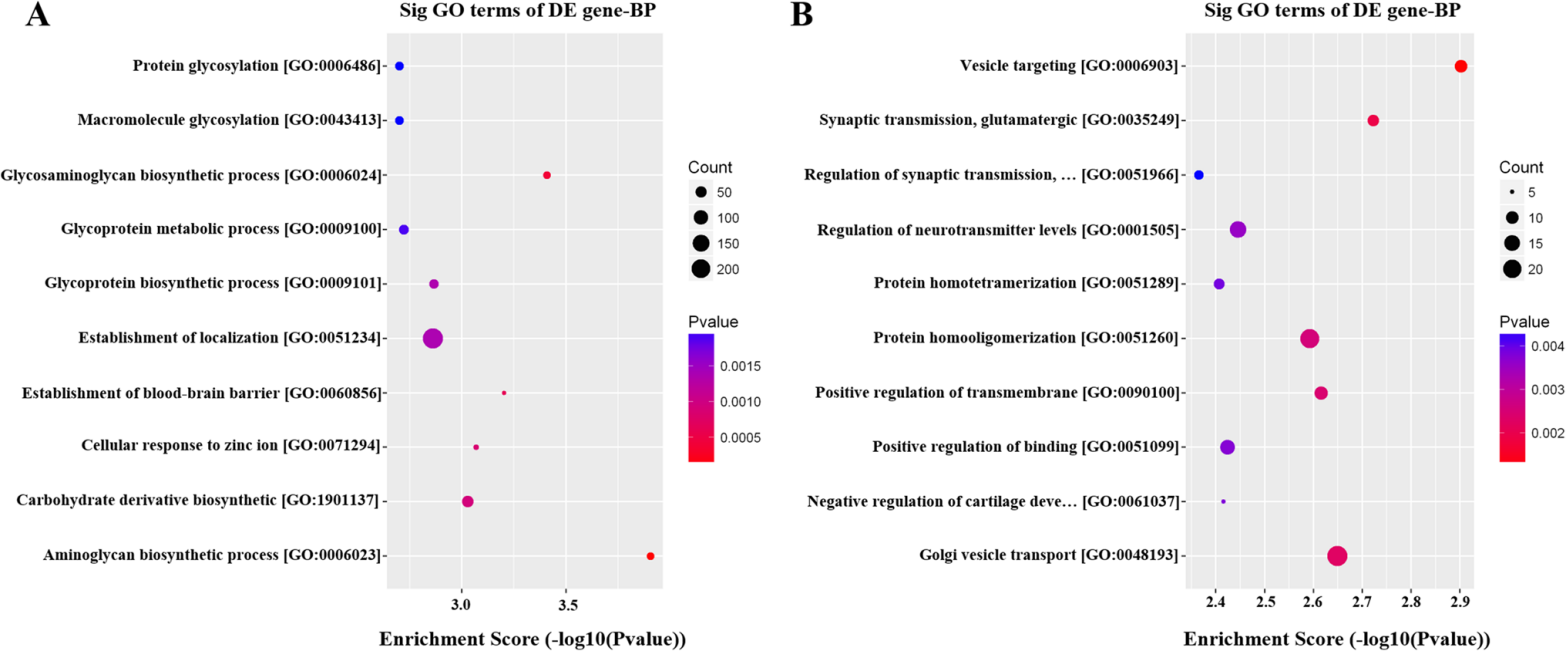
Gene Ontology (GO) enrichment analyses. (A) Upregulated genes and (B) downregulated genes

The Gene Ontology (GO) (http://www.geneontology.org) and Kyoto Encyclopedia of Genes and Genomes (KEGG) pathway enrichment analysis (http://www.genome.jp/kegg) were performed to explore potential functions of differentially expressed genes and correlated pathways. Dot plots (Fig. 2) showed the results of GO enrichment. The upregulated mRNAs (Fig. 2A) include protein glycosylation, macromolecule glycosylation, glycosaminoglycan biosynthetic process, glycoprotein metabolic process, glycoprotein biosynthetic process, establishment of localization, establishment of blood–brain barrier, cellular response to zinc ion, carbohydrate derivative biosynthetic, and aminoglycan biosynthetic process, whereas biological process of downregulated (Fig. 2B) mRNAs includes vesicle targeting, synaptic transmission, glutamatergic, regulation of synaptic transmission, regulation of neurotransmitter levels, protein homo tetramerization, protein homo oligomerization, positive regulation of transmembrane, positive regulation of blinding, negative regulation of cartilage, and golgi vesicle transport.

**Fig. 3:**
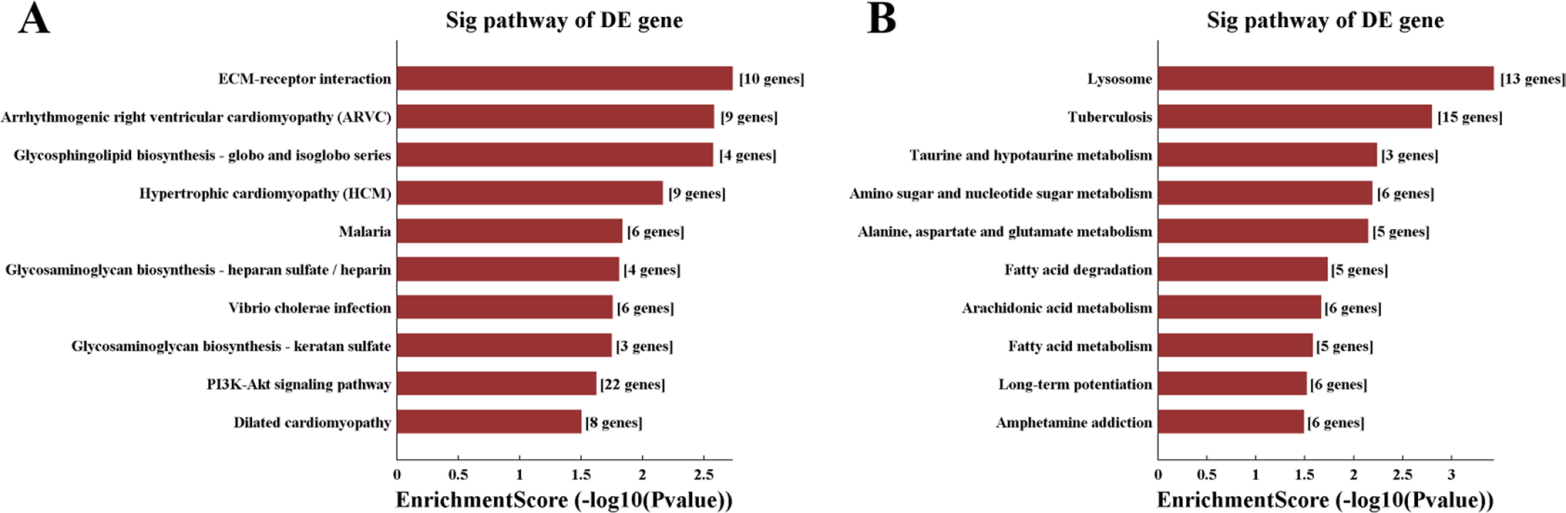
Kyoto Encyclopedia of Genes and Genomes (KEGG) pathway analyses. (A) KEGG pathway enrichment analyses showing 10 pathways enriched among upregulated genes. (B) KEGG pathway enrichment analyses showing 10 pathways enriched among downregulated genes. DE, differentially expressed

The KEGG pathway enrichment analyses showed that 10 pathways were significantly enriched in upregulated genes (Fig. 3A), including ECM-receptor interaction, arrhythmogenic right ventricular cardiomyopathy (ARVC), glycosphingolipid biosynthesis-globo and isoglobo series, hypertrophic cardiomyopathy (HCM), malaria, glycosaminoglycan biosynthesis-heparan sulfate/heparin, vibrio cholerae infection, glucosaminoglycan biosynthesis-keratan sulfate, PI3K-Akt signaling pathway, and dilated cardiomyopathy. Moreover, ten pathways were enriched in downregulated genes (Fig. 3B), including lysosome, tuberculosis, taurine and hypotaurine metabolism, amino sugar and nucleotide sugar metabolism, alanine, aspartate and glutamate metabolism, fatty acid degradation, arachidonic acid metabolism, fatty acid metabolism, long-term potentiation, and amphetamine addiction.

### Real-time quantitative PCR validation

**Fig. 4:**
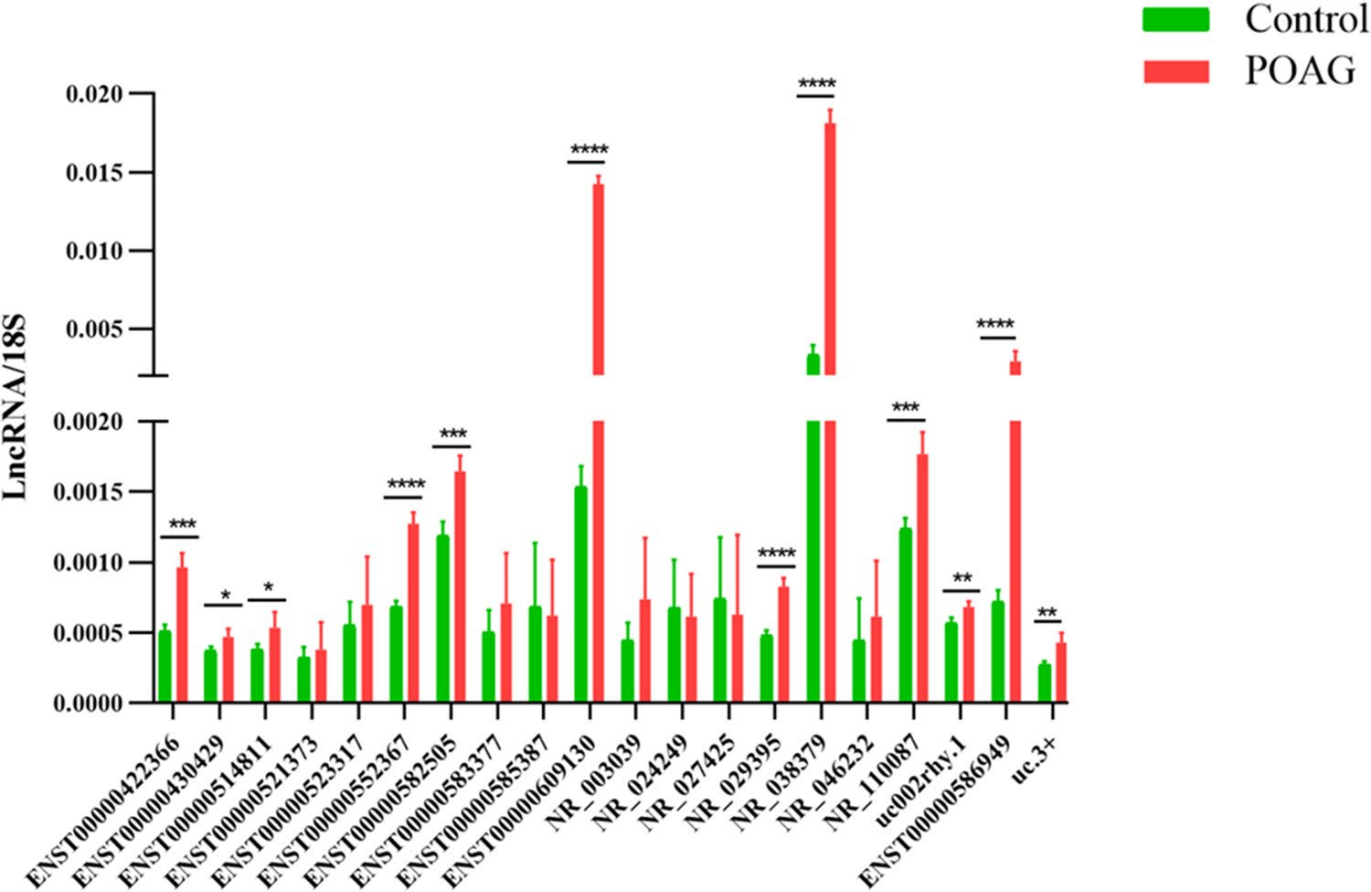
Real-time quantitative PCR validation of 20 lncRNAs in TM samples from control group and POAG group. Data are expressed as means ± SD

To confirm the microarray analysis results and identify potential related lncRNA for development of POAG, 20 lncRNAs (Table 2) that had highly significant P values (P < 0.01), large fold changes (> 3.0), false discovery rate (< 0.025), and stated positive correlation with POAG-relevant mRNA are chosen and listed in Table 3. Their expression was assessed by individual RT-qPCR assays using the same samples from the initial microarray analysis (Fig. 4). Consistent with the microarray results, ENST00000422366, ENST00000430429, ENST00000514811, ENST00000552367, ENST00000582505, ENST00000609130, NR_029395, NR_038379, NR_110087, uc002rhy.1, ENST00000586949, and uc.3 + were significantly upregulated (***P = 0.0002, *P = 0.0191, *P = 0.0431, ****P < 0.0001, ***P = 0.0009, ****P < 0.0001, ****P < 0.0001, ****P < 0.0001, ***P = 0.0009, **P = 0.0062, ***P = 0.0004, **P = 0.006, respectively) in the POAG group (Fig. 4). Otherwise, there were no significant differences in expression levels of ENST00000521373, ENST00000523317, ENST00000583377, ENST00000585387, NR_003039, NR_024249, NR_027425, and NR_046232 in trabecular meshwork samples between control and POAG groups (Fig. 4). As shown in Fig. 4, most genes (12/20) were consistent with the direction of changes acquired by microarray analysis, confirming the validity of the microarray data.

**Table 2:** Primer sequences of 20 lncRNAs

Primer name	Primer sequence
18S rRNA	F:5′CAGCCACCCGAGATTGAGCA3′ R:5′ TAGTAGCGACGGGCGGTGTG3′
ENST00000422366	F:5′ CTCAGGACACCTCCCGTTGC 3′ R:5′ TGGGCATCCGTTTGTTTGACT 3′
ENST00000430429	F:5′ GCCACAATAGCAGGAAACCTA 3′ R:5′ GTCTTGCAGATGGGAGACCA 3′
ENST00000514811	F:5′ CTGAAAGGAGCCCCTTGACA 3′ R:5′ CGTCTGACCAATGAAAACCGT 3′
ENST00000521373	F:5′ GAGTGTGGGGTGGGTCTGAA 3′ R:5′ GCACAGGACAGGCGATTTGA 3′
ENST00000523317	F:5′ TTGCCGCTGTTGGATGTCA 3′ R:5′ CCTGACTTTGCTTTCTCTGACCT 3′
ENST00000552367	F:5′ ACCTTACCTTGTCTTGCCCG 3′ R:5′ GAGATCACGAGCCGCACTC 3′
ENST00000582505	F:5′ACTGAAGCGACCTTTCCTCG3′ R:5′ CGAGGTGCTCCGGGAATC3′
ENST00000583377	F:5′ CAGTGGCTCAATCATAGCTCACT 3′ R:5′ AGTAACTGGAACCACAGGCACA 3′
ENST00000585387	F:5′ CCACCAGACAGAGCAGGATG 3′ R:5′ TCTTCCACAAGGGATGGAATG 3′
ENST00000609130	F:5′ TTGAGCCTTACGCAGAGGTCT 3′ R:5′ TTGGTGGGTAAAGAGGGTGGA 3′
NR_003039	F:5′ GCCTCCTTCCACAACTCTCA 3′ R:5′ AGGCTGAGTCTCCGAGTGAA 3′
NR_024249	F:5′ AGCCCAGAAGCCATCGTGTC 3′ R:5′ TGATCCCAGCCCGGCATA 3′
NR_027425	F:5′ GTGCCACAACGGGAATCTTG 3′ R:5′ ATCAAATTGGTGCCTGGGGTA 3′
NR_029395	F:5′ AACAGAGCAACAGCAAGTACAT 3′ R:5′ CTGGGAACCTATGAACATTCT 3′
NR_038379	F:5′ TACTTTGTGCCAGGGCCTTAT 3′ R:5′ TCTTTCCCAACTAAACCGTGAG 3′
NR_046232	F:5′ GAGCACTGAGGACCCTTCTTG 3′ R:5′ AGCCCACTGACACCTTGACTT 3′
NR_110087	F:5′ AGCAGTCCACCCCTGGCTG 3′ R:5′ CCAAATAGCTTGCAGTGCTCTGT 3′
uc002rhy.1	F:5′ GAAAGTCGGATGCTGAAGATG 3′ R:5′GCAGGTAGAGTAGAGTCTGAGGG 3′
ENST00000586949	F:5′ GAAGCAGGAAAAGACAGTCTCTA 3′ R:5′ CAGTCTGGTGTACAAGGCAGAA 3′
uc.3 +	F:5′ATTTGCATAACCCAACCCC3′ R:5′ CGATGTCGTCCTAATTCACC3′

*F*, forward; *R*, reverse

**Table 3:** A collection of lncRNAs detected using microarray in POAG patients

Seqname	Gene Symbol	Type	Source	Chrom	Fold Change	P-value
ENST00000422366	HCG25	noncoding	GENCODE	chr6	6.295	0.000
ENST00000430429	AC0988 28.2	noncoding	GENCODE	chr2	8.012	0.001
ENST00000514811	CTB-174D11.2	noncoding	GENCODE	chr5	3.062	0.000
ENST00000521373	CTB-43E15.2	noncoding	GENCODE	chr5	3.462	0.000
ENST00000523317	RP11-513H8.1	noncoding	GENCODE	chr8	9.207	0.000
ENST00000552367	RP11-290L1.3	noncoding	GENCODE	chr12	3.587	0.002
ENST00000582505	RP11-180P8.1	noncoding	GENCODE	chr17	4.507	0.007
ENST00000583377	RP11-848P1.5	noncoding	GENCODE	chr17	8.063	0.000
ENST00000585387	RP11-47L3.1	noncoding	GENCODE	chr17	3.514	0.000
ENST00000609130	RP11-127 5H24.2	noncoding	GENCODE	chr7	3.421	0.000
NR_00 3039	GLY CAM1	noncoding	RefSeq	chr12	3.403	0.000
NR_02 4249	FAM 86C2P	noncoding	RefSeq	chr11	3.578	0.001
NR_02 7425	FAM66D	noncoding	RefSeq	chr8	5.053	0.000
NR_02 9395	IGLL3P	noncoding	RefSeq	chr22	3.317	0.000
NR_03 8379	LOC 554,206	noncoding	RefSeq	chr16	4.230	0.001
NR_04 6232	LINC 01,298	noncoding	RefSeq	chr8	3.719	0.001
NR_11 0087	LOC10 1,927,497	noncoding	RefSeq	chr7	5.512	0.000
uc002rhy.1	AK125 769	noncoding	UCSC_ knowngene	chr2	6.741	0.000
ENST00000586949	RP11-879F14.2	noncoding	GENCODE	chr18	3.112	0.000
uc.3 +	uc.3	noncoding	UCR	chr1	5.113	0.000

**Fig. 5:**
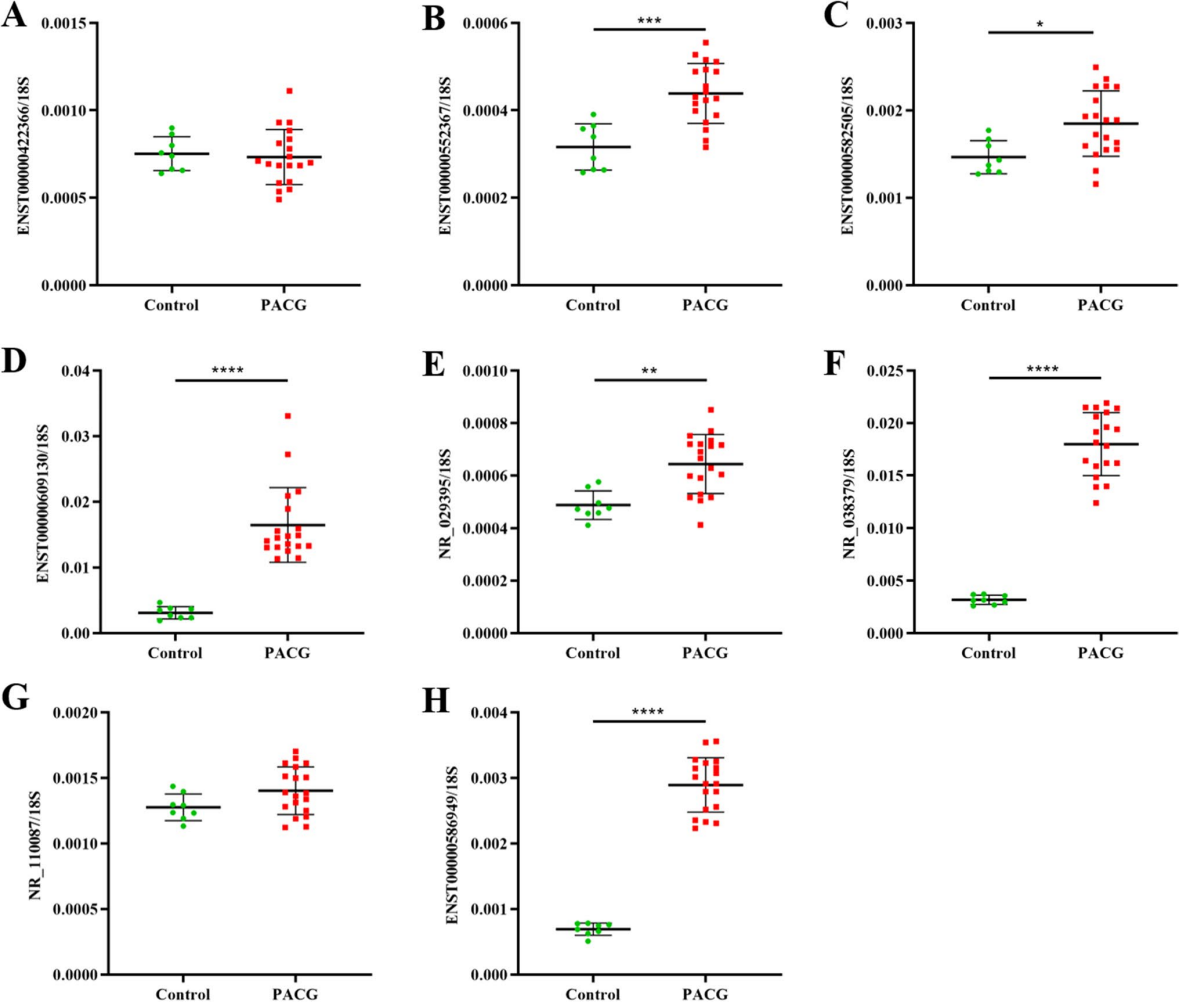
Real-time quantitative PCR showing expression of ENST00000422366 (A), ENST00000552367 (B), ENST00000582505 (C), ENST00000609130 (D), NR_029395 (E), NR_038379 (F), NR_110087 (G), and ENST00000586949 (H) in TM of control group and POAG group. Data are expressed as means ± SD

To further confirm the results, more samples (8 cases of normal trabecular meshwork samples and 19 cases of POAG tissue samples) were collected and we confirmed that the expression of ENST00000552367, ENST00000582505, ENST00000609130, NR_029395, NR_038379, and ENST00000586949 was significantly upregulated (***P = 0.0001, *P = 0.0113, ****P < 0.0001, **P = 0.001, ****P < 0.0001, ****P < 0.0001, respectively) (Fig. 5B, C, D, E, F, H). However, ENST00000422366 and NR_110087 show no significant difference expression levels in trabecular meshwork samples between control and POAG groups (Fig. 5A, G).

## Discussion

Long non-coding RNA demonstrates ~ tenfold lower abundance than mRNAs in a population of cells and characterized as tissue-specific [17]. In addition to higher tissue specificity, lncRNAs are characterized by higher developmental stage specificity [18]. Recent recognition that lncRNAs function in various aspects of cell biology has caused increasing attention on their potential contribution towards diseases etiology [19]. In the glaucoma research area, previous studies have indicated that there is a relationship between lncRNA and glaucoma. Lili Xie et al. [12] identified lncRNAs T267384, ENST00000607393, and T342877 may be potential biomarkers for POAG diagnosis and ENST00000607393 might be a new therapeutic target for trabecular meshwork calcification based on clinical tissues. J. Zhao et al. [11] concluded that lncRNA ANRIL attenuated oxidative injury of human TM cells and activated the mTOR and MEK/ERK pathways, possibly through downregulation of miR-7. Y. Xu et al. [20] found that downregulation of lncRNA GAS5 may maintain retinal ganglion cell survival in glaucoma through the activation of TGF-β pathway to promote cell proliferation and differentiation. Haibo Li et al. [21] provided evidence that lncRNA-MALAT1 could inhibit RGC apoptosis in glaucoma through activation of the PI3K/Akt signaling pathway. Shen W. et al. [22] established that oxidative stress–induced lncRNA-RP11-820 plays a key role in regulating the miR-3178/MYOD1/ECM axis in HTMCs. Moreover, our previous work [23] had proved that knockdown of lncRNA NR_003923 in human Tenon’s capsule fibroblast cells (HTFs) inhibited TGF-β-induced cell migration, proliferation, fibrosis, and autophagy and overexpression of IL22RA1 enhanced HTF migration and proliferation. Therefore, NR_003923 and IL22RA1 might contribute to glaucoma progression.

However, detailed analyses on expression profiling of lncRNAs in TM of POAG patients have not yet to be reported. Glaucoma has a complex pathogenesis and its symptoms are associated with the long-term intraocular pressure and damage, as well as apoptosis of retinal ganglion cells caused by various pathological factors [24]. Among these multiple factors, IOP, the major risk one for the development and progression of glaucoma, is closely associated with TM tissue [25]. The TM is a series of fenestrated beams and sheets of the extracellular matrix and is responsible for draining the aqueous humor from the eye via the anterior chamber. Therefore, TM tissue plays a crucial role in the development and progression of glaucoma [26]. It would be more convincing to collect TM tissue rather than other ocular tissues for further microarray analyses. This study is the largest comparison of lncRNA expression in the TM of normal controls and POAG patients reported to date.

According to these results, lncRNAs ENST00000552367, ENST00000582505, ENST00000609130, NR_029395, NR_038379, and ENST00000586949 have high expression in TM of POAG. These findings could point us to potential routes of therapy beyond that of intraocular pressure–lowing medications or surgery. The data help clarify the processes that eventually cause POAG and in so doing improve the prospects of a better understanding of this disease process, along with more rational approaches for the development of therapies. However, the sample size from our study was relatively small and these samples only came from the Chinese population. These results may serve as bases for further researches in this area. The functions of these lncRNAs should be further verified through experiment in vivo and vitro.

## Conclusions

We conclude that lncRNAs ENST00000552367, ENST00000582505, ENST00000609130, NR_029395, NR_038379, and ENST00000586949 may play essential roles in the development of POAG.
